# MMP14 as a central mediator of TGF-β1−induced extracellular matrix remodeling in graves’ orbitopathy

**DOI:** 10.3389/fendo.2025.1623842

**Published:** 2025-07-22

**Authors:** Xing Wang, Jing Lu, Yuxia He, Qinxin Shu, Yuxin Lin, Wenqi Su, Peng Wang

**Affiliations:** Department of Ophthalmology, The First Affiliated Hospital of Chongqing Medical University, Chongqing Key Laboratory for the Prevention and Treatment of Major Blinding Eye Diseases, Chongqing, China

**Keywords:** graves’ orbitopathy (GO), matrix metalloproteinase 14 (MMP14), fibrosis, extracellular matrix, tissue remodeling

## Abstract

**Background:**

Graves’ orbitopathy (GO) is an autoimmune orbital disorder characterized by chronic inflammation and aberrant extracellular matrix (ECM) remodeling, leading to progressive fibrosis. Recent studies implicate matrix metalloproteinase−14 (MMP14) in ECM degradation and tissue remodeling, yet its precise role in GO remains unclear.

**Design and methods:**

Orbital adipose/connective tissues specimens were obtained from GO patients (stratified into type I and type II based on clinical classification) and non−GO controls. High−throughput RNA sequencing identified differentially expressed genes, focusing on MMP−related transcripts. MMP14 expression was quantified by immunohistochemistry and Western blotting, correlating its levels with fibrotic grade. Primary orbital fibroblasts (OFs) isolated from GO and control subjects were cultured and stimulated with TGF−β1. Quantitative real−time PCR and Western blot assays evaluated MMP14 and fibroblast activation markers (α−SMA, COL1A1, CTGF). Transcriptomic profiling of TGF−β1–treated OFs and a scratch wound assay further assessed the effect of the MMP14 inhibitor NSC−405020 on cellular motility.

**Results:**

GO type II tissues demonstrated a significant upregulation of MMP14, which correlated positively with fibrosis severity. GO− derived OFs exhibited higher basal and TGF−β1–induced MMP14 and fibrotic marker expression compared to controls. Transcriptomic analysis revealed activation of ECM–receptor interaction, PI3K−Akt, and MAPK signaling pathways enriched for MMP−associated genes. Pharmacologic inhibition of MMP14 attenuated TGF−β1–induced fibrotic markers and reduced OFs migration.

**Conclusion:**

These findings indicate that MMP14 is a central mediator in GO fibrotic remodeling, highlighting its potential as a therapeutic target to alleviate orbital fibrosis. Further mechanistic studies are needed to clarify MMP14’s role in GO progression.

## Highlights

- GO type II tissues exhibited a marked upregulation of MMP14, with expression levels positively correlating with fibrosis severity.
- MMP14 is a crucial mediator in the fibrotic remodeling process of GO, promoting ECM reorganization and fibroblast activation in response to TGF−β1.
- Targeting MMP14 may be a novel therapeutic strategy to alleviate orbital fibrosis in GO.

## Introduction

Graves’ orbitopathy (GO) represents one of the most prevalent adult orbital disorders, affecting approximately 20% of patients with thyroid dysfunction (1). The condition manifests through various clinical sequelae including proptosis, diplopia, and ocular surface irritation, with severe cases progressing to corneal ulceration, compressive optic neuropathy, and potentially permanent visual impairment (2). As an autoimmune pathology, GO is characterized by chronic orbital inflammation and extensive tissue remodeling within the orbital compartment. Clinically, GO is stratified into type I (adipose-predominant) and type II (extraocular muscle-predominant) variants (3), representing distinct yet potentially overlapping pathophysiological processes with divergent prognostic implications. Type II patients typically experience more severe manifestations accompanied by significant orbital fibrosis. Disease progression is mediated by complex interactions between infiltrating immune cells and resident orbital fibroblasts (OFs) (4), with the thyroid-stimulating hormone receptor (TSHR) and insulin-like growth factor 1 receptor (IGF1R) established as principal autoantigenic targets (5). Teprotumumab, a monoclonal antibody directed against IGF-1R, has demonstrated remarkable therapeutic efficacy, representing a paradigm shift in GO management (6). OFs function as critical cellular mediators, secreting diverse proinflammatory and profibrotic factors including IL-1β, IL-2, IL-6, CXCL8, IL-10, COX2, CCL2, CCL5, TGF-β (7, 8), as well as IFN-γ and TNF-α (9). This inflammatory microenvironment orchestrates OF trans-differentiation into myofibroblasts, culminating in pathological extracellular matrix (ECM) deposition and progressive fibrosis—hallmark features underlying orbital tissue expansion, proptosis, and compressive optic neuropathy in advanced disease (10).

Matrix metalloproteinases (MMPs) constitute a diverse enzyme family responsible for selective degradation and remodeling of extracellular matrix (ECM) components (11). Among this proteolytic repertoire, MMP1, MMP2, MMP9, and notably MMP14 have been implicated as critical mediators of inflammatory processes, tissue reorganization, and repair mechanisms (12). Dysregulated MMP activity disrupts physiological ECM turnover dynamics, potentially accelerating tissue fibrosis and driving pathological progression in GO (13–15). MMP14 (membrane type 1-MMP), a transmembrane metalloproteinase, exerts multifaceted functions extending beyond matrix degradation to include modulation of cellular signaling cascades (16). This membrane-anchored protease orchestrates diverse physiological and pathological processes, including tissue invasion, neovascularization, and immunomodulatory responses (17). Consequently, elucidating MMP14’s precise contribution to GO pathophysiology may identify novel therapeutic targets for disease intervention.

The intricate interplay between inflammatory mediators, aberrant ECM accumulation, and MMP14-mediated tissue remodeling represents a fundamental axis in GO pathogenesis, highlighting these molecular pathways as compelling candidates for therapeutic targeting. We hypothesize that MMP14 functions as both a necessary and sufficient mediator of TGF-β1-driven extracellular matrix remodeling in orbital fibroblasts, thereby constituting a potentially viable therapeutic target for attenuating fibrotic progression in GO.

## Subjects and methods

### Samples and reagents

Orbital adipose and connective tissue specimens were harvested from 14 patients with confirmed GO undergoing therapeutic orbital decompression procedures. All patients exhibited biochemical euthyroidism at the time of surgical intervention. GO patients were stratified according to the 1991 Nunery classification system (3): Type I subjects predominantly manifested orbital adipose tissue expansion with mild to moderate extraocular muscle involvement, without restrictive myopathy or diplopia; Type II subjects demonstrated significant extraocular muscle enlargement with resulting restrictive myopathy and diplopia within the central 20° visual field. Control specimens were procured from seven non-GO patients undergoing enucleation for uveal melanoma (UM), with inclusion criteria stipulating absence of extraocular extension or metastatic disease. Comprehensive clinical and demographic characteristics of the study cohort are detailed in **Table 1**.

**Table 1:** Clinical features of patient samples used in this study.

Age (Years)	Gender (M/F)	Smoking (Y/N)	Duration of GO (Years)	Disease	Previous GO treatment	Proptosis (R/L, mm)	CAS	GO severity assessment	Surgery performed
GO patients									
54	F	N	2	GO type I	None	19/18	0	III	Decompression
52	M	Y	1.5	GO type II	GCs	21/20	0	VI	Decompression
62	M	Y	2	GO type II	GCs	20/19	1	VI	Decompression
55	F	N	1	GO type I	None	18/19	2	III	Decompression
48	M	N	1.5	GO type I	GCs	20/21	0	III	Decompression
50	M	Y	2.5	GO type II	None	21/20	2	IV	Decompression
40	F	N	3	GO type I	None	22/21	0	III	Decompression
47	M	Y	1.75	GO type II	GCs	22/20	1	VI	Decompression
53	M	Y	1	GO type II	None	19/20	3	VI	Decompression
63	F	N	3	GO type II	None	19/18	1	IV	Decompression
37	F	N	2	GO type I	None	21/21	0	III	Decompression
44	F	N	2.5	GO type I	None	22/21	1	III	Decompression
41	M	N	1	GO type I	None	22.5/21	2	III	Decompression
60	M	Y	0.5	GO type II	GCs	19/21	2	VI	Decompression
Non- GO control patients									
65	M	N	–	UM	–	–	–	–	Enucleation
50	M	Y	–	UM	–	–	–	–	Enucleation
57	F	N	–	UM	–	–	–	–	Enucleation
53	F	N	–	UM	–	–	–	–	Enucleation
42	M	N	–	UM	–	–	–	–	Enucleation
55	M	Y	–	UM	–	–	–	–	Enucleation
48	M	Y	–	UM	–	–	–	–	Enucleation

M, male, F, female; Y, yes; N, no; GO, thyroid-associated ophthalmopathy; UM, uveal melanoma; R/L, right or left eyes; CAS, clinical ac-tivity score; GCs, glucocorticoids. NOSPECS classification (0 = no symptoms or signs; I = only signs, no symptoms; II = soft tissue involvement; III = proptosis; IV = EOM involvement; V = corneal involvement; VI = sight loss, due to optic nerve involvement).

Exclusion criteria encompassed administration of systemic or local glucocorticoid therapy within three months preceding tissue acquisition. Disease severity and inflammatory activity were assessed using the standardized NOSPECS classification system and seven-point clinical activity score (CAS) as established by the European Group on Graves’ Orbitopathy (EUGOGO) (18, 19). Written informed consent was obtained from all study participants. This investigation was conducted in accordance with the ethical principles outlined in the Declaration of Helsinki and received approval from the Institutional Review Board of the First Affiliated Hospital of Chongqing Medical University (approval number 2023-30, January 11, 2023).

Dulbecco’s Modified Eagle’s Medium (DMEM, #C11965500BT), fetal bovine serum (FBS, #10270-106-1), penicillin and streptomycin (#15140122), 0.25% trypsin/EDTA (#25200072), and phosphate-buffered saline (PBS, #C10010500BT) (all reagents from Gibco Laboratories, New York, NY, USA). Dimethyl sulfoxide (DMSO, #WL064, Meiluncell), TGF-β1 (R&D Systems, Minneapolis, MN, USA, #240-B-010), MMP-14 inhibitor (NSC-405020, #HY-15827, MCE);

TaKaRa MiniBEST Universal RNA Extraction Kit (#9767), PrimeScript RT Master Mix (#RR036B) and TB Green Premix Ex Taq II (#RR820B) (all from TaKaRa, Dalian, China); Radioimmunoprecipitation Assay (RIPA, #P0013B, Beyotime), Protein-free rapid blocking solution (#G2052, Servicebio), Enhanced BCA Protein Assay kit (#P0010, Beyotime), 5×protein loading buffer (Boster). Primary antibody: α-SMA (#19245S), COL1A1 (#39952S), CTGF (#86641S), GAPDH (#5174S) (all from Cell Signaling Technology, Boston, MA, USA); MMP14 (#AF0212), PI3K p85 alpha (#AF6241), Phospho-PI3K p85 alpha (Tyr607, #AF3241), pan-AKT1/2/3 (#AF6261), Phospho-AKT1/2/3 (Ser473, #AF0016), β-actin (#AF7018) (all from Affinity Biosciences); DyLight 488 Goat anti-Rabbit IgG (H + L) Secondary Antibody (#A23220, Abbkine), Goat anti-Rabbit IgG HRP Conjugated Secondary Antibody (#CW0103S, CWBIO). HRP-conjugated anti-rabbit secondary antibody (#DM-001, ProteinSimple, San Jose, CA, USA).

### Immunohistochemistry

Formalin-fixed, paraffin-embedded tissue specimens were sectioned at 3-μm thickness and subjected to a standardized immunohistochemical protocol. Deparaffinization was performed using xylene, followed by gradient rehydration through absolute ethanol series. Antigen retrieval was accomplished using EDTA buffer (pH 9.0) under optimized conditions. Following triple rinses with phosphate-buffered saline (PBS), endogenous peroxidase activity was quenched by incubating sections in 3% hydrogen peroxide solution for 25 minutes at ambient temperature under light protection. After subsequent PBS washing, nonspecific binding sites were blocked using 3% bovine serum albumin (BSA) applied dropwise onto sections with 30-minute incubation at room temperature. Primary anti-MMP14 antibody was applied to tissue sections with overnight incubation at 4°C in a humidified chamber. Following thorough PBS washing, sections were incubated with horseradish peroxidase-conjugated goat anti-rabbit IgG secondary antibody (Servicebio, Wuhan, Hubei, China) for 50 minutes at room temperature. Immunoreactivity was visualized using diaminobenzidine (DAB) chromogen with precisely timed development (45 seconds), followed by hematoxylin counterstaining. Processed sections underwent dehydration through graded alcohols and were permanently mounted using neutral mounting medium. Quantitative immunohistochemical analysis was performed using ImageJ software (National Institutes of Health, Bethesda, MA, USA), with integrated optical density (IOD) normalized to measured area serving as the semiquantitative metric for MMP14 expression levels.

### RNA sequencing

Total RNA was isolated from primary orbital fibroblast cultures utilizing the TaKaRa MiniBEST Universal RNA Extraction Kit following the manufacturer’s optimized protocol. RNA quality and integrity were assessed prior to downstream applications. Subsequently, high-quality RNA samples were processed for cDNA library construction using the NEBNext^®^ Ultra™ RNA Library Prep Kit for Illumina platform through a systematic workflow including mRNA isolation, fragmentation, and double-stranded cDNA synthesis according to the manufacturer’s specifications. Preliminary quality assessment and quantification of the resultant cDNA libraries were performed using Qubit 3.0 fluorometric quantitation, with a stringent quality control threshold requiring effective library concentrations exceeding 10 nM. Libraries meeting these rigorous quality parameters were sequenced on the Illumina NovaSeq 6000 platform employing paired-end 150 bp (PE150) sequencing chemistry to ensure comprehensive transcriptomic coverage and robust read depth for differential expression analysis.

### Primary cultures of OFs

Orbital adipose/connective tissue specimens were meticulously dissected to remove fascial elements and vascular structures prior to processing. The resulting tissue was finely minced into approximately 1-mm³ fragments and established as explant cultures in 10-cm tissue culture dishes containing high-glucose Dulbecco’s Modified Eagle Medium (DMEM) supplemented with 20% fetal bovine serum (FBS) and 1% penicillin/streptomycin. Following fibroblast outgrowth from tissue explants and attainment of 80-90% confluence, adherent cells were enzymatically dissociated using 0.25% trypsin-EDTA solution and subcultured. Established orbital fibroblast (OF) cultures were subsequently maintained in standard proliferation medium comprising DMEM supplemented with 10% FBS and 1% penicillin/streptomycin under standard culture conditions (37°C, 5% CO_2_, humidified atmosphere). All experimental procedures were performed using OFs between passages 3 and 7, with a minimum of three biological replicates per experimental condition to ensure reproducibility.

### RNA extraction and real-time polymerase chain reaction

Total RNA was isolated from cultured OFs using the TaKaRa MiniBEST Universal RNA Extraction Kit according to the manufacturer’s standardized protocol. RNA concentration and purity were spectrophotometrically determined prior to reverse transcription. Complementary DNA (cDNA) synthesis was performed using the PrimeScript RT Master Mix under optimized reaction conditions. Quantitative real-time PCR was conducted on a Roche LightCycler 480 system (Roche Diagnostics, Basel, Switzerland) employing TB Green Premix Ex Taq II reagent with gene-specific primers. Oligonucleotide primer sequences utilized for target gene amplification are comprehensively detailed in **Table 2**. Glyceraldehyde-3-phosphate dehydrogenase (GAPDH) expression served as the endogenous reference control for normalization of target gene expression.

**Table 2:** RT-qPCR primer sequences.

Genes	Sequences (5’-3’)
MMP14	F: GGGTTCCTGGCTCATGCCTA R: GTGACCCTGACTTGCTTCCATAA
GAPDH	F: TTGCCATCAATGACCCCTT R: CGCCCCACTTGATTTTGGA

F, forward; R, reverse.

### Western blotting

Cell and tissue lysates were prepared using a protein extraction kit (KeyGEN, Nanjing, China; #KGP250, #KGP950), and protein concentrations were quantified using a BCA assay kit (Beyotime, Shanghai, China; #P0010). Due to equipment constraints, two distinct Western blotting methodologies were employed. A subset of samples was analyzed with an automated capillary electrophoresis system (Simple Western system with Compass software; ProteinSimple, San Jose, CA, USA; Version 5.0.0) using Wes Separation Capillary Cartridges (covering molecular weight ranges of 12–230 kDa and 66–440 kDa; #SM-W004 and #SM-W008, ProteinSimple). In parallel, other samples were prepared by boiling for 5–6 minutes after the addition of 5× protein loading buffer to achieve denaturation. These samples were then separated via SDS-PAGE (loading 30 μg of protein for cells and 60 μg for eyeball tissue) and transferred onto polyvinylidene difluoride (PVDF) membranes (Millipore, USA). The membranes were blocked at room temperature for 30 minutes using a protein-free rapid blocking solution, followed by overnight incubation at 4°C with the primary antibody. After washing with Tris-HCl-buffered saline containing Tween 20 (TBST), membranes were incubated with horseradish peroxidase (HRP)-conjugated secondary antibodies for 1–2 hours at room temperature. Protein bands were visualized using a gel imaging system (Thermo Fisher, USA) and quantified with ImageJ software (National Institutes of Health, Bethesda, MA, USA).

### Wound healing assay

Confluent orbital fibroblast monolayers in 6-well plates were mechanically disrupted using sterile pipette tips to create uniform linear wounds. Initial wound dimensions were measured immediately post-disruption to establish baseline values. Culture medium was then replaced with fresh medium containing MMP-14 inhibitor NSC-405020 (100 μM) and TGF-β1 (10 ng/mL), followed by incubation for 24 and 48 hours. Wound closure progression was documented via phase-contrast microscopy (Leica Microsystems GmbH, 4× magnification) with quantitative analysis of wound width reduction.

### Statistical analysis

All experiments were conducted with biological triplicates using samples from distinct individuals, with technical duplicates for each condition. Data are expressed as mean ± standard deviation. Statistical analyses were performed using GraphPad Prism v10 (GraphPad Software) employing one-way ANOVA with *post-hoc* tests. Statistical significance was defined as p<0.05.

## Results

### Transcriptomic profiling and enrichment analysis of orbital tissues in TAO

**Figure 1:**
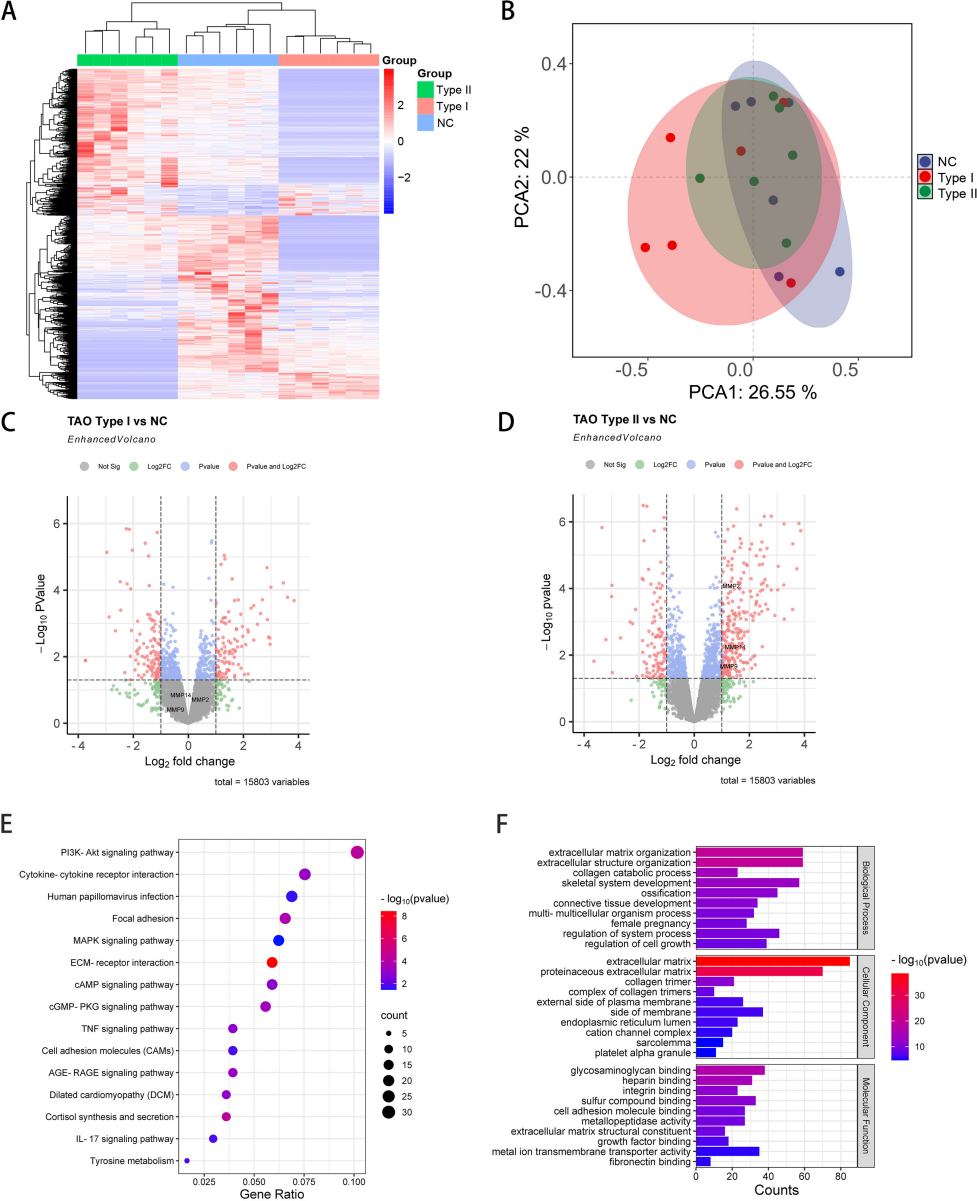
Comprehensive transcriptomic analysis of orbital tissues from GO and NC patients. **(A)** Hierarchical clustering heatmap depicting differentially expressed transcripts across GO and NC orbital tissue specimens (n=6 per group). Color intensity indicates magnitude of expression deviation from the mean. **(B)** Principal component analysis (PCA) plot illustrating transcriptional variance and sample clustering based on the first two principal components, demonstrating distinct molecular signatures among experimental groups. **(C)** Volcano plot representation of differentially expressed transcripts in GO type I versus NC comparison. Red and blue dots represent significantly upregulated and downregulated genes, respectively (fold change ≥2.0, P<0.05). **(D)** Volcano plot analysis of differentially expressed transcripts in GO type II versus NC comparison, highlighting substantial transcriptional reprogramming in this disease phenotype. **(E)** KEGG pathway enrichment analysis of differentially expressed genes in GO type II orbital tissues. Bubble size corresponds to gene count, while color intensity indicates statistical significance. **(F)** Gene Ontology (GO) biological process enrichment analysis of differentially expressed genes in GO type II, with bar length representing statistical significance (-log10 P-value).

Comprehensive transcriptomic analysis was performed on orbital adipose/connective tissue specimens obtained from demographically matched normal control (NC) subjects and patients with distinct GO phenotypes (type I and type II). RNA sequencing identified 15,803 transcripts across all samples (**Figure 1A**). Principal component analysis revealed distinct transcriptional signatures among experimental groups, with the first two components (PC1 and PC2) accounting for 26.55% and 22% of total variance, respectively (**Figure 1B**). Differential expression analysis identified 229 significantly modulated transcripts between GO type I and NC specimens (106 upregulated, 123 downregulated; fold change ≥2.0, P<0.05) (**Figure 1C**). The GO type II versus NC comparison yielded 405 differentially expressed genes, comprising 279 upregulated transcripts—notably including matrix metalloproteinases MMP14, MMP9, and MMP2—and 126 downregulated transcripts (fold change ≥2.0, P<0.05) (**Figure 1D**). Based on these findings, subsequent analyses focused on GO type II versus NC comparisons. KEGG pathway enrichment analysis revealed significant involvement of 54 signaling cascades, predominantly including PI3K-Akt signaling, MAPK signaling, and ECM-receptor interaction pathways (**Figure 1E**). Complementary Gene Ontology analysis demonstrated significant enrichment in biological processes governing extracellular matrix remodeling, cell-matrix adhesion, collagen fibril organization, and cytoskeletal regulation (**Figure 1F**).

### GO orbital connective tissues exhibit elevated MMP14 levels correlated with fibrosis severity

**Figure 2:**
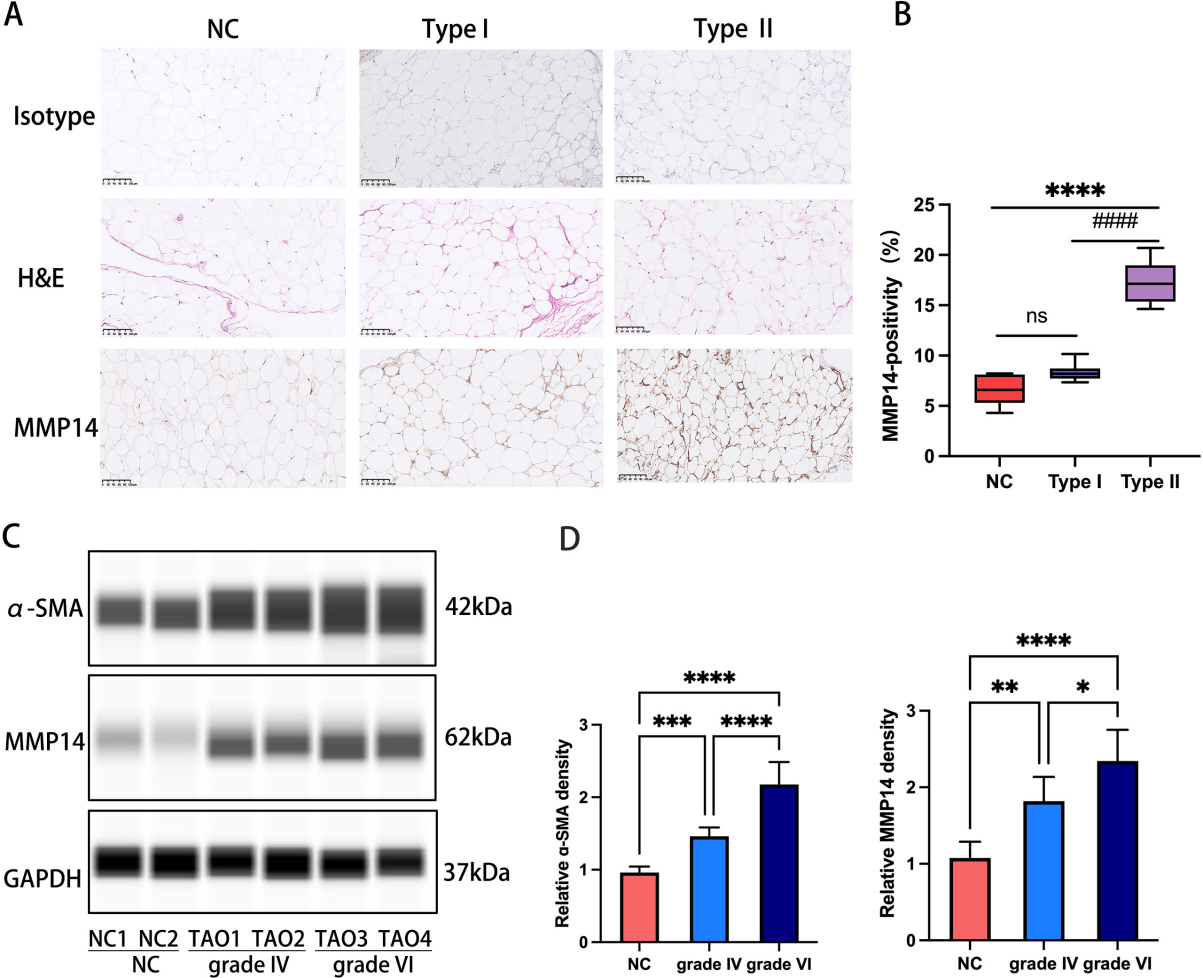
MMP14 expression in orbital tissues correlates with fibrotic severity in GO **(A)** Representative immunohistochemical detection of MMP14 in orbital tissue sections from NC, GO type I, and GO type II patients. MMP14 immunoreactivity appears as yellowish-brown chromogenic signal. Scale bars: 100 μm. **(B)** Semi-quantitative analysis of MMP14 immunopositivity across experimental groups (NC: n=5; GO type I: n=7; GO type II: n=7). **(C)** Representative immunoblot analysis of MMP14 and α-SMA protein expression in orbital adipose/connective tissue specimens from normal controls and GO patients with varying fibrotic grades. **(D)** Densitometric quantification of protein expression normalized to GAPDH. Values represent mean ± SD from three independent experiments. Statistical significance was determined by one-way ANOVA with *post-hoc* analysis: ns, not significant; ####p<0.0001 compared to NC; *p<0.05, **p<0.01, ***p<0.001, ****p<0.0001 between indicated groups.

Immunohistochemical evaluation of orbital adipose/connective tissue specimens demonstrated distinct MMP14 expression patterns across experimental cohorts. Normal control (NC) and GO type I tissues exhibited minimal MMP14 immunoreactivity, whereas GO type II specimens displayed pronounced MMP14 upregulation (P<0.0001; **Figures 2A, B**). Complementary Western blot analysis of protein extracts from orbital adipose tissues stratified by clinical severity (NC, GO grade IV, and GO grade VI) revealed progressive elevation of both α-smooth muscle actin (α-SMA) and MMP14 protein levels in GO specimens compared to controls. Notably, expression intensity of both markers demonstrated a significant positive correlation with histopathological fibrotic grade (**Figures 2C, D**), suggesting mechanistic involvement in disease progression.

### TGF-β1 induces MMP14 upregulation in orbital fibroblasts from GO patients

**Figure 3:**
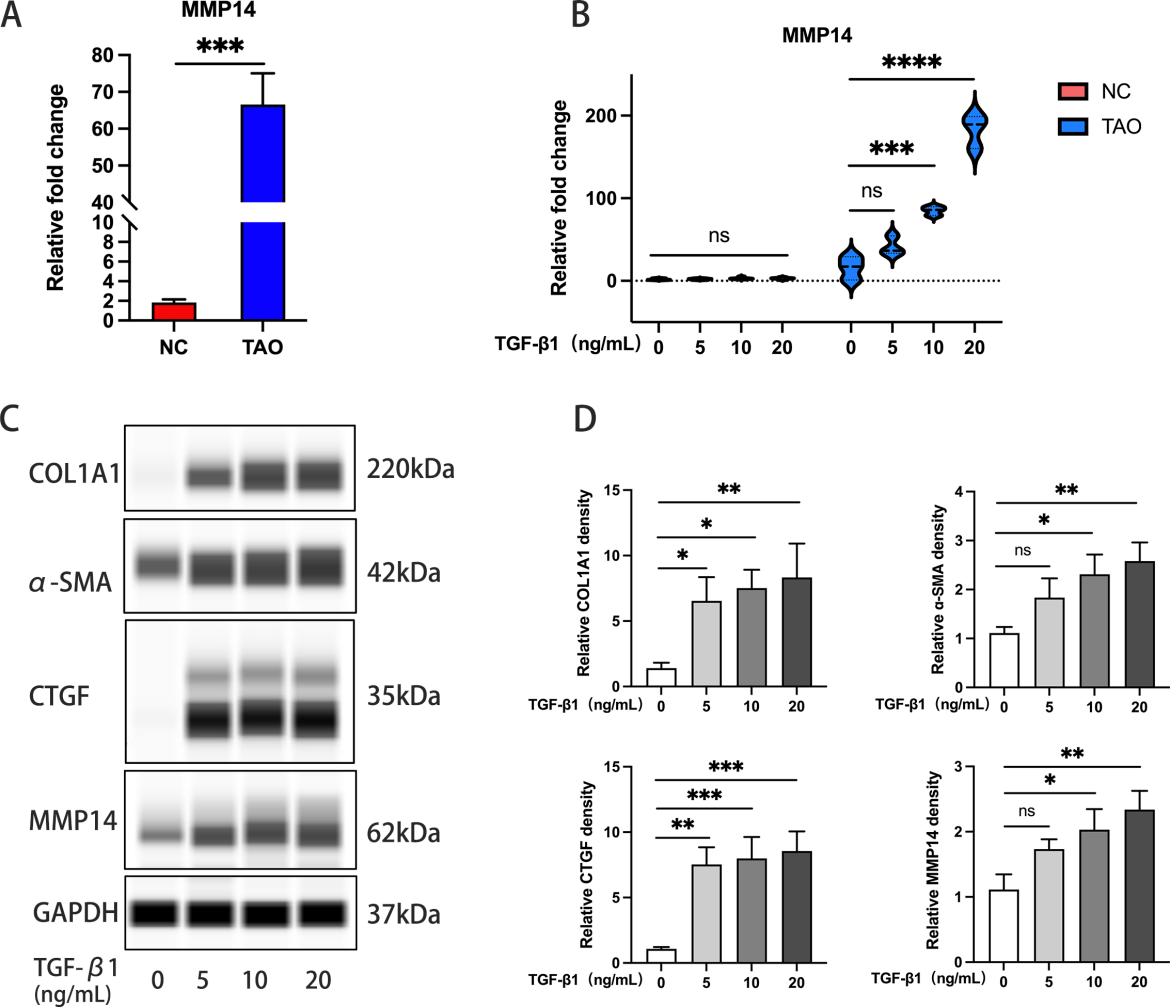
TGF-β1 induces MMP14 upregulation in OFs from GO patients. **(A)** Comparative analysis of baseline MMP14 transcript levels in OFs isolated from Graves’ ophthalmopathy patients (GO, n=10) and normal controls (NC, n=7). ****p<0.0001 vs. control group. **(B)** Quantitative PCR analysis of MMP14 expression in OFs following TGF-β1 stimulation at varying concentrations. GO-derived cells (n=3) demonstrated concentration-dependent upregulation compared to normal control cells (n=5). **(C)** Representative immunoblots showing protein expression of fibrotic markers (COL1A1, α-SMA, CTGF) and MMP14 across experimental conditions following TGF-β1 treatment. **(D)** Densitometric quantification of protein expression normalized to GAPDH (n=3). Data presented as mean ± standard deviation. Statistical significance was determined by one-way ANOVA with *post-hoc* analysis: ns, not significant; *p<0.05, **p<0.01, ***p<0.001, ****p<0.0001 between indicated groups.

Baseline transcriptional analysis revealed significantly elevated MMP14 expression in primary OFs derived from GO patients compared to those isolated from normal control subjects (**Figure 3A**). To establish optimal experimental parameters, CCK-8 viability assays were employed to determine appropriate TGF-β1 concentrations and exposure times, while qPCR was used to assess fibrotic marker expression changes. Based on these preliminary studies, concentrations of 5, 10, and 20 ng/mL TGF-β1 were selected for subsequent experiments (**Supplementary Figures 1A, B**). Differential responsiveness to TGF-β1 stimulation was observed between GO and control-derived OFs. GO fibroblasts exhibited a concentration-dependent upregulation of MMP14 at 10 and 20 ng/mL TGF-β1, whereas control fibroblasts showed no significant expression changes under identical conditions (**Figure 3B**). Furthermore, extended exposure (48 hours) to TGF-β1 induced significant transcriptional activation of multiple fibrotic markers, including COL1A1, CTGF, α-SMA, and MMP14, specifically in GO-derived orbital fibroblasts (**Figures 3C, D**). These findings collectively suggest that TGF-β1-mediated MMP14 induction contributes significantly to the pathological fibrotic response observed in GO orbital fibroblasts.

### Comprehensive transcriptomic profiling reveals MMP14 as a mediator in TGF-β1-induced fibrotic response

**Figure 4:**
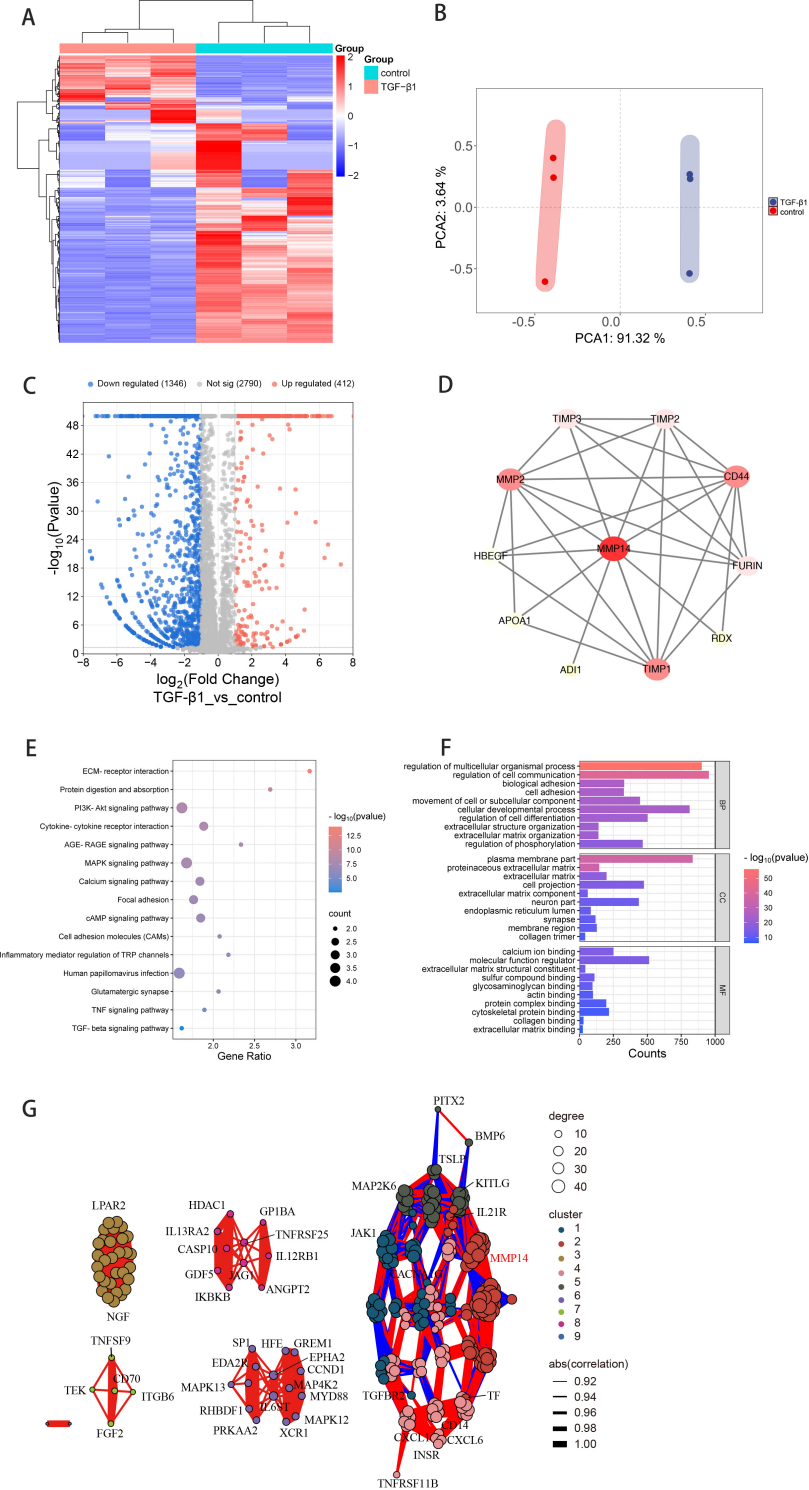
Transcriptomic analysis reveals MMP14-associated regulatory networks in TGF-β1-stimulated GO orbital fibroblasts. **(A)** Hierarchical clustering heatmap depicting differentially expressed transcripts after intersection with the MMP gene database in GO orbital fibroblasts (n=3) with or without TGF-β1 stimulation (10 ng/mL, 24h). Color intensity reflects standardized expression values (z-scores). **(B)** Principal component analysis of normalized RNA-sequencing data demonstrating distinct transcriptional profiles between treatment conditions. First two principal components (PC1: 91.32%, PC2: 3.64% of variance) reveal clear separation between TGF-β1-treated and control samples. **(C)** Volcano plot visualization of differential gene expression. Red and blue dots represent significantly upregulated (412) and downregulated (1,346) transcripts, respectively (fold change ≥2.0, P<0.05). **(D)** Protein-protein interaction network analysis illustrating functional connections between MMP14 and fibrosis-associated proteins. Node size reflects interaction degree; edge thickness indicates confidence score of protein associations. **(E)** KEGG pathway enrichment analysis presented as a bubble chart. Bubble size corresponds to gene count; color intensity represents significance level (-log10 P-value) of enriched pathways in TGF-β1-treated versus control conditions. **(F)** Gene Ontology enrichment analysis showing top significantly modulated biological processes following TGF-β1 treatment. Bar length indicates statistical significance (-log10 P-value). **(G)** Correlation network visualization integrating MMP14 with 255 genes from enriched signaling pathways. Edge color and thickness represent correlation strength and directionality.

To elucidate the molecular mechanisms underlying TGF-β1-mediated fibrosis in Graves’ ophthalmopathy, orbital fibroblasts (OFs) isolated from three independent GO patients were exposed to 10 ng/mL TGF-β1 for 24 hours prior to transcriptome analysis by high-throughput RNA sequencing. This systematic approach identified 7,229 significantly differentially expressed genes (DEGs) compared to untreated controls. Cross-referencing these DEGs with the MMP gene database yielded 1,758 MMP-associated transcripts (**Figure 4A**). Principal component analysis demonstrated distinct transcriptional profiles between TGF-β1-treated and control samples, with the first two principal components (PC1 and PC2) accounting for 91.32% and 3.64% of the observed variance, respectively (**Figure 4B**). Differential expression analysis visualized through volcano plotting revealed 412 significantly upregulated and 1,346 downregulated genes (fold change ≥2.0, P<0.05; **Figure 4C**).

Protein-protein interaction (PPI) network mapping illuminated extensive functional connections between MMP14 and numerous proteins implicated in fibrotic processes and signal transduction pathways (**Figure 4D**). KEGG pathway enrichment analysis identified 54 significantly modulated signaling networks, with particularly strong enrichment in extracellular matrix-receptor interaction, PI3K-Akt signaling, MAPK signaling cascade, and cell adhesion molecule pathways (**Figure 4E**). Complementary Gene Ontology analysis revealed significant enrichment in biological processes critical to fibrogenesis, including cell adhesion, extracellular matrix organization, and collagen trimer formation (**Figure 4F**). To further characterize MMP14’s role in these regulatory networks, we constructed a correlation network integrating MMP14 with 255 selected genes derived from the enriched pathways, providing insights into potential functional relationships governing fibrotic transformation in GO (**Figure 4G**).

### MMP14 inhibition attenuates TGF-β1-induced fibrotic responses in GO orbital fibroblasts

**Figure 5:**
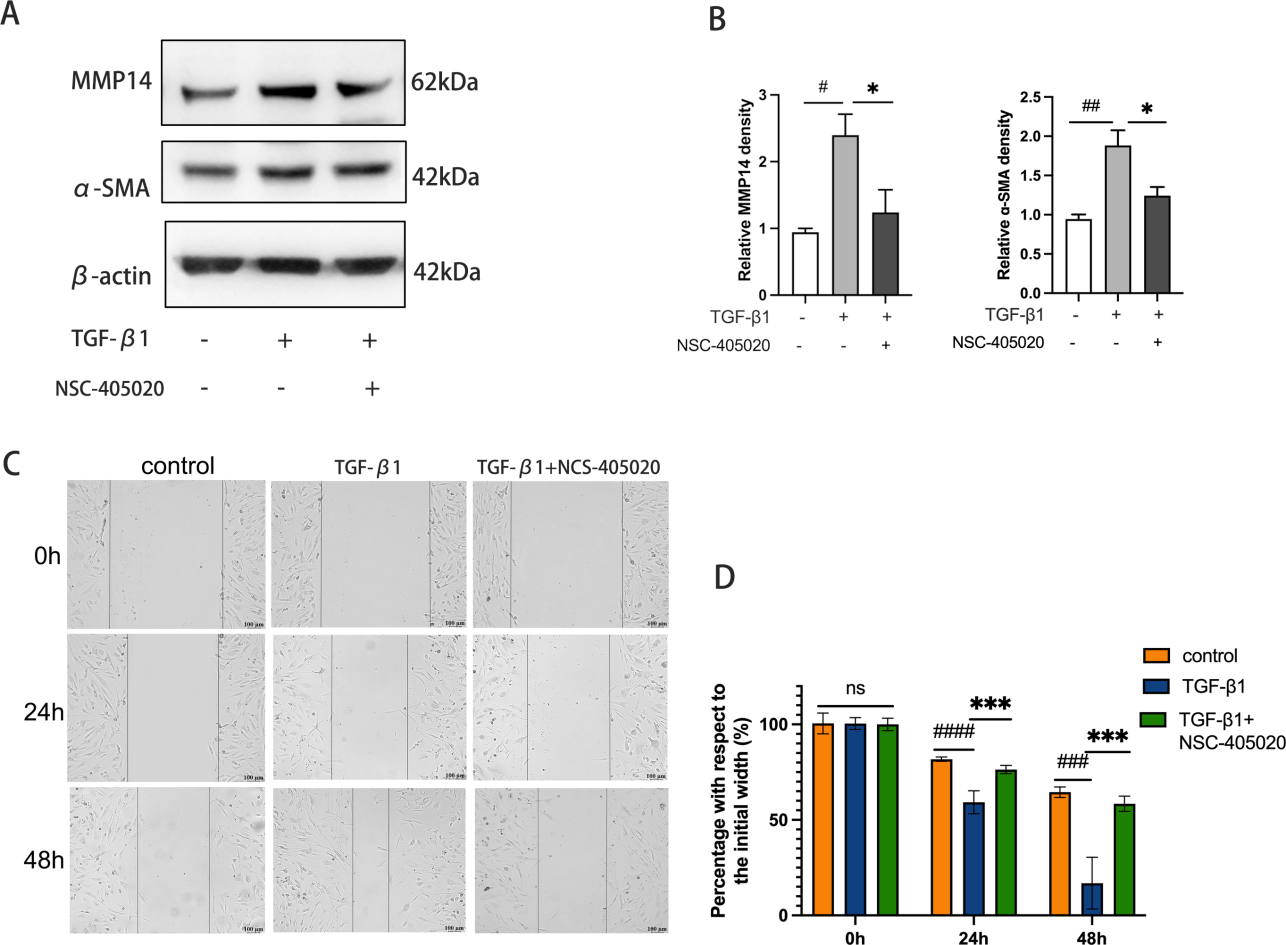
Pharmacological Inhibition of MMP14 Suppresses TGF-β1-induced fibrotic response and migratory capacity in orbital fibroblasts. **(A)** Representative immunoblots demonstrating protein expression of α-SMA and MMP14 in orbital fibroblasts following treatment with TGF-β1 (10 ng/mL) and/or MMP14 inhibitor NCS-405020 (100 μM). β-actin served as loading control. **(B)** Densitometric quantification of protein expression normalized to β-actin across experimental conditions (n=3). Results demonstrate significant attenuation of TGF-β1-induced protein expression by MMP14 inhibition. **(C)** Representative phase-contrast micrographs of scratch wound healing assay showing orbital fibroblast migration at indicated time points following treatment with TGF-β1 (10 ng/mL) and/or MMP14 inhibitor (100 μM). Scale bar = 100 μm. **(D)** Quantitative analysis of wound closure rates across treatment conditions (n=3). Values represent percentage of initial wound area closed at specified time points. Data are presented as mean ± standard deviation from triplicate experiments. Statistical significance was determined by one-way ANOVA with *post-hoc* analysis: ns, not significant; #p<0.05, ##p<0.01, ###p<0.001, ####p<0.0001 compared to control group; *p<0.05, ***p<0.001 between indicated experimental groups.

To investigate the functional significance of MMP14 in fibrotic pathogenesis, orbital fibroblasts derived from GO patients were treated with the selective MMP14 inhibitor NCS-405020 in the presence of TGF-β1. Immunoblot analysis revealed that pharmacological inhibition of MMP14 substantially suppressed TGF-β1-induced expression of both MMP14 and the myofibroblast marker α-SMA (**Figures 5A, B**), indicating attenuation of the fibrotic phenotype. The impact on cellular functionality was further assessed using scratch wound migration assays. While TGF-β1 stimulation significantly enhanced orbital fibroblast motility and wound closure capacity, concurrent treatment with NCS-405020 markedly impaired this migratory response. Temporal quantification of wound gap measurements confirmed significant dose-dependent inhibition of cellular migration following MMP14 inhibition (**Figures 5C, D**). These findings collectively demonstrate that MMP14 activity is essential for TGF-β1-mediated fibroblast activation and migration, key processes in the pathological tissue remodeling characteristic of GO.

## Discussion

Ongoing investigations into matrix metalloproteinases (MMPs) in GO pathophysiology (20), continue to advance our understanding toward the development of targeted therapeutic interventions that selectively modulate specific MMP activities to mitigate aberrant tissue remodeling and ameliorate clinical manifestations. In the current investigation, we employed comprehensive transcriptome profiling through high-throughput RNA sequencing of orbital connective tissue specimens obtained from GO type I patients, GO type II patients, and non-GO control subjects. This systematic approach facilitated the identification of disease-specific transcriptional signatures and elucidated the molecular architecture underlying GO progression.

Comparative transcriptomic analysis revealed a distinct molecular profile in GO type II tissues, characterized by significant upregulation of 279 transcripts—notably including MMP14—compared to both GO type I specimens and control tissues. Subsequent functional bioinformatics interrogation through KEGG pathway and Gene Ontology enrichment analyses demonstrated significant activation of biological processes governing extracellular matrix homeostasis, particularly ECM–receptor interactions, matrix organizational dynamics, and collagen assembly pathways. These molecular signatures align with and extend recent literature highlighting extracellular matrix dysregulation as a central mechanistic determinant in GO pathogenesis and disease progression (15, 21).

MMP14 emerges as a critical mediator of extracellular matrix remodeling and fibrosis through its multifunctional capacity. This transmembrane metalloproteinase not only directly degrades structural ECM components (22), but also functions as an activator of other MMPs (23), a modulator of cellular signaling networks (24), a regulator of cell phenotype and behavior (25), and a modifier of ECM protein bioactivity. These diverse functions operate under precise spatiotemporal control in a context-dependent manner across tissues and pathological states (26). Elucidating MMP14’s comprehensive role in matrix dynamics and fibrogenesis is fundamental to developing innovative therapeutic strategies targeting fibrotic disorders. Recent advances have expanded MMP14’s functional repertoire beyond matrix degradation to include immunomodulatory functions, particularly in facilitating M0 macrophage infiltration into affected tissues (27). Furthermore, MMP14 serves as a sophisticated regulator of cytokine bioavailability within the extracellular milieu. Karsdal and colleagues demonstrated that MMP14 specifically cleaves the latency-associated peptide (LAP) of TGF-β1, liberating biologically active TGF-β1 from sequestration within ECM reservoirs (28). Our investigations reveal that MMP14 expression in GO-derived orbital fibroblasts increases proportionally with fibrotic severity, suggesting a cell-specific role in disease progression. This pattern may represent an adaptive compensatory mechanism by which orbital fibroblasts attempt to counterbalance excessive matrix accumulation. Paradoxically, this putative homeostatic response may ultimately destabilize the delicate equilibrium of ECM turnover, thereby accelerating pathological fibrosis. Within orbital fibroblasts, locally activated TGF-β1 initiates canonical SMAD2/3 phosphorylation cascades, as confirmed by our phosphoproteomic analyses. This signaling pathway establishes a self-amplifying regulatory circuit wherein TGF-β1 stimulation enhances MMP14 expression through SMAD-responsive elements within the MMP14 promoter region (29). This reciprocal regulatory mechanism acquires particular pathophysiological significance in thyroid-associated ophthalmopathy (TAO), where orbital fibroblasts reside within a TGF-β1-enriched microenvironment. The concomitant elevation of both MMP14 protein and phosphorylated SMAD2/3 in type II TAO tissues provides compelling evidence supporting this mechanistic relationship.

Our *in vitro* experimental findings further demonstrate that orbital fibroblasts derived from GO patients exhibit constitutively elevated MMP14 expression compared to control subjects, suggesting these cells exist in a “primed” or “pre-activated” state with heightened susceptibility to fibrotic stimuli—a phenotypic alteration likely induced by chronic exposure to the inflammatory microenvironment characteristic of GO (30). Moreover, exogenous TGF-β1 stimulation substantially augmented MMP14 expression in GO-derived orbital fibroblasts while simultaneously upregulating established fibrotic markers including COL1A1, CTGF, and α-SMA. These data collectively indicate that TGF-β1 orchestrates GO fibrotic progression through coordinated regulation of multiple fibrosis-associated genes (31).

Comprehensive transcriptomic profiling of TGF-β1-stimulated GO orbital fibroblasts further illuminated the molecular networks governing fibrotic transformation. Cross-reference analysis with the MMP gene database identified 1,758 MMP-associated transcripts significantly modulated by TGF-β1 treatment, underscoring the extensive influence of this cytokine on MMP regulatory networks. Pathway enrichment analyses revealed significant perturbation of several signaling cascades—notably ECM–receptor interaction, PI3K–Akt, and MAPK pathways—while Gene Ontology analyses demonstrated enrichment in biological processes governing cell adhesion, extracellular matrix organization, and collagen assembly. This systems-level transcriptional reprogramming reinforces MMP14’s position as a central regulatory node in TGF-β1-mediated fibrogenesis in GO.

MMP14 further influences intercellular communication and cell-matrix interactions by modulating the functional activity of diverse membrane-anchored and extracellular proteins (32). Illustrating this regulatory complexity, in human extravillous trophoblasts, leptin promotes cell invasion by upregulating MMP14 expression—a process effectively neutralized by PI3K/Akt pathway inhibition (33). Similarly, in SiHa cervical cancer cells, MMP14 induction is abrogated by pharmacological inhibition of MAPK/ERK signaling using PD98059 or U0126 (34). In mammary epithelial cells, MMP14 directly interacts with integrin β1 to regulate MAPK signaling, thereby facilitating tumor cell invasiveness (35). These diverse examples illuminate the intricate functional interrelationships connecting MMP14, ECM receptors, PI3K–Akt signaling, and MAPK cascades, highlighting the need for comprehensive mechanistic investigations of MMP14 biology in GO pathogenesis.

The application of NCS-405020, a selective MMP14 inhibitor, provided compelling functional evidence substantiating MMP14’s mechanistic role in GO-associated fibrotic pathology. Pharmacological antagonism of MMP14 activity not only attenuated TGF-β1-induced upregulation of α-SMA—a canonical myofibroblast marker—but also significantly impeded orbital fibroblast migration in wound healing assays. This simultaneous suppression of fibrotic marker expression and cellular motility upon MMP14 blockade strongly indicates that selective targeting of this metalloproteinase represents a promising therapeutic strategy to mitigate orbital tissue fibrosis in GO. Corroborating our findings, previous investigations across diverse fibrotic disorders have demonstrated MMP14’s functional significance in pathological matrix remodeling. In systemic sclerosis, siRNA-mediated silencing of MMP14 expression in dermal fibroblasts substantially diminished TGF-β1-induced transcription of fibrotic genes (36). In cardiac fibroblasts, TGF-β1 stimulation upregulates furin expression, which subsequently activates MMP14; notably, pharmacological inhibition of furin significantly reduces MT1-MMP/MMP-2 activation and impairs fibroblast migration (37). Furthermore, in human keratinocytes, targeted depletion of MMP14 through RNA interference markedly attenuates TGF-β1-stimulated cellular migration—an effect mechanistically linked to suppression of JNK signaling pathway activation (38). Collectively, these cross-disciplinary observations reinforce MMP14’s central role in TGF-β1-mediated fibrotic processes and cellular phenotypic transformation.

Despite these significant insights, several methodological limitations warrant acknowledgment. First, our experimental paradigm focused exclusively on isolated orbital fibroblasts, thereby precluding comprehensive analysis of potential interactions with immunologically active cellular populations and the influence of the complex orbital microenvironment that characterizes *in vivo* pathogenesis. Second, the precise molecular mechanisms and signaling networks through which MMP14 orchestrates orbital fibroblast activation and fibrotic transformation require further detailed elucidation. Third, the absence of validated experimental animal models that faithfully recapitulate the orbital manifestations of GO substantially constrains evaluation of MMP14-targeted interventions in an intact physiological system.

Translational implementation of MMP14 inhibition in GO presents additional challenges related to ocular drug delivery. Achieving therapeutic concentrations within the anatomically restricted orbital connective tissues would likely necessitate localized administration approaches—such as periocular or sub-Tenon injection of advanced controlled-release formulations (including biodegradable hydrogels or nanoparticulate delivery systems)—to maximize target-site bioavailability while minimizing systemic exposure. However, the orbit’s extensive vascular and lymphatic drainage networks introduce potential concerns regarding unintended redistribution to adjacent ocular structures, necessitating meticulous dose optimization and comprehensive preclinical toxicological assessment to safeguard retinal integrity, trabecular outflow facility, and extraocular muscle functionality.

## Conclusion

Our comprehensive investigations demonstrate that elevated MMP14 expression is integrally associated with pathological extracellular matrix remodeling and progressive fibrosis in GO. The pronounced reduction in fibrotic marker expression and impaired fibroblast motility following selective MMP14 inhibition highlights this metalloproteinase’s therapeutic potential as an intervention target in GO. While additional mechanistic investigations are essential to fully elucidate MMP14’s regulatory roles, the current work establishes a fundamental framework for developing targeted therapeutic strategies to counteract fibrotic progression in GO. Future research endeavors should focus on (1): dissecting the intricate molecular networks governing MMP14 expression and activation in orbital fibroblasts (2); developing advanced delivery technologies specifically tailored to the unique anatomical and physiological characteristics of the orbital microenvironment; and (3) establishing well-defined therapeutic parameters for MMP14 inhibition that maximize anti-fibrotic efficacy while preserving essential physiological matrix remodeling functions in orbital tissues.

## Data Availability

The datasets presented in this study can be found in online repositories. The names of the repository/repositories and accession number(s) can be found in the article/**Supplementary Material**.
