# Supplementary material for: MMP14 as a central mediator of TGF-β1−induced extracellular matrix remodeling in graves’ orbitopathy
Source: Front Endocrinol (Lausanne). 2025 Jul 22;16:1623842. doi: 10.3389/fendo.2025.1623842 (PMC12321542; doi:10.3389/fendo.2025.1623842)
Supplement: Supplementary file 1 [file DataSheet1.docx]

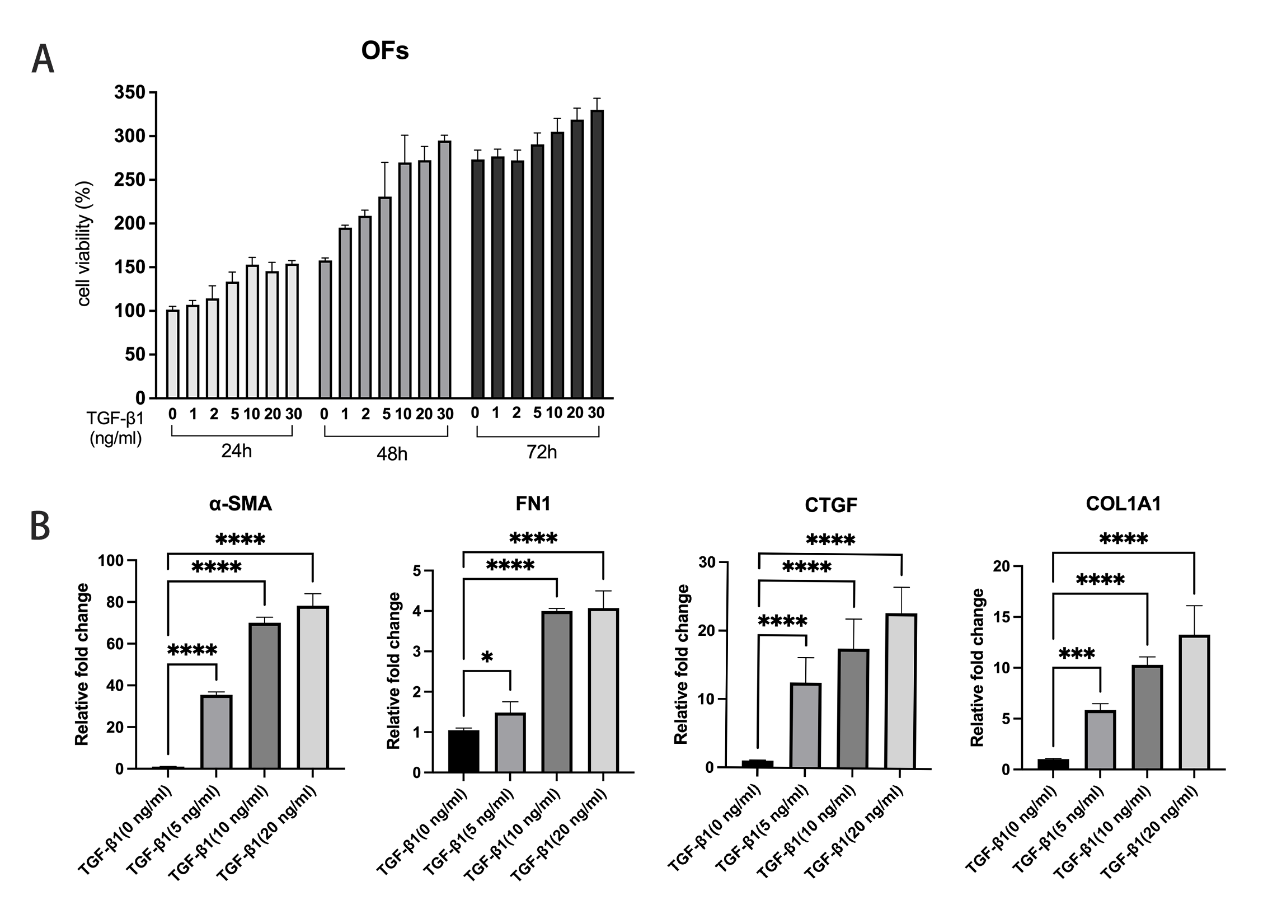


**Supplementary Figure 1 (S1). Noncytotoxic concentration of TGF-β1**

A. GO OFs were treated with increasing concentrations of TGF-β1 (0, 1, 2, 5, 10, 20, and 30 μM) for 24 h, 48 h, and 72 h. Cell viability is presented as the percentage relative to the viability of untreated cells.

B. mRNA levels of α-SMA, FN1, CTGF, and COL1A1. Each graph is obtained from the mean ± SD of repeated experiments from 6 GO cell samples.

The data are expressed as the mean ± standard deviation (SD) of triplicate replicates. *p < 0.05, ***p < 0.001，and ****p<0.0001 compared with untreated group; no mark denotes no statistical significance; assessed by one-way ANOVA.
